# Specific transcriptional programs differentiate ICOS from CD28 costimulatory signaling in human Naïve CD4^+^ T cells

**DOI:** 10.3389/fimmu.2022.915963

**Published:** 2022-09-05

**Authors:** Casimiro Luca Gigliotti, Elena Boggio, Francesco Favero, Danny Incarnato, Claudio Santoro, Salvatore Oliviero, Josè Maria Rojo, Silvia Zucchelli, Francesca Persichetti, Gianluca Baldanzi, Umberto Dianzani, Davide Corà

**Affiliations:** ^1^ Department of Health Sciences, University of Piemonte Orientale, Novara, Italy; ^2^ Interdisciplinary Research Center of Autoimmune Diseases (IRCAD), University of Piemonte Orientale, Novara, Italy; ^3^ CAAD - Center for Translational Research on Autoimmune and Allergic Disease, Novara, Italy; ^4^ Department of Molecular Genetics, Groningen Biomolecular Sciences and Biotechnology Institute (GBB), University of Groningen, Groningen, Netherlands; ^5^ Dipartimento di Scienze della Vita e Biologia dei Sistemi, Università di Torino, Torino, Italy; ^6^ Italian Institute for Genomic Medicine (IIGM), Torino, Italy; ^7^ Centro de Investigaciones Biológicas Margarita Salas, Consejo Superior de Investigaciones Cientificas (CSIC), Madrid, Spain; ^8^ Department of Translational Medicine, University of Piemonte Orientale, Novara, Italy; ^9^ Biochemical Chemistry, “Maggiore della Carità” University Hospital, Novara, Italy

**Keywords:** human CD4^+^ T cells, T-cell receptor, ICOS, CD28, RNA sequencing

## Abstract

Costimulatory molecules of the CD28 family play a crucial role in the activation of immune responses in T lymphocytes, complementing and modulating signals originating from the T-cell receptor (TCR) complex. Although distinct functional roles have been demonstrated for each family member, the specific signaling pathways differentiating ICOS- from CD28-mediated costimulation during early T-cell activation are poorly characterized. In the present study, we have performed RNA-Seq-based global transcriptome profiling of anti-CD3-treated naïve CD4^+^ T cells upon costimulation through either inducible costimulator (ICOS) or CD28, revealing a set of signaling pathways specifically associated with each signal. In particular, we show that CD3/ICOS costimulation plays a major role in pathways related to STAT3 function and osteoarthritis (OA), whereas the CD3/CD28 axis mainly regulates p38 MAPK signaling. Furthermore, we report the activation of distinct immunometabolic pathways, with CD3/ICOS costimulation preferentially targeting glycosaminoglycans (GAGs) and CD3/CD28 regulating mitochondrial respiratory chain and cholesterol biosynthesis. These data suggest that ICOS and CD28 costimulatory signals play distinct roles during the activation of naïve T cells by modulating distinct sets of immunological and immunometabolic genes.

## Introduction

Activation of naïve T helper (Th) cells and their consequent differentiation require three signals: the first one is delivered by the T-cell receptor (TCR) upon recognition of the antigenic peptide presented by the appropriate major histocompatibility complex (MHC) molecule; the second one is generated by T-cell costimulatory receptors engaged by their ligands expressed on antigen-presenting cells (APCs); and the third one is generated by cytokines available in the microenvironment. When the first signal is triggered in the absence of the second one, it initiates a genetic program resulting in anergy or apoptosis (1, 2).

The best-known T helper (Th) cell costimulatory receptor is CD28, which is constitutively expressed by all Th cells. CD28 binds to B7.1 (CD80) and B7.2 (CD86) on APCs, and promotes cell proliferation and cytokine secretion (3–5).

Another Th costimulatory receptor is inducible costimulator (ICOS, also called CD278), which belongs to the CD28 family. ICOS is selectively expressed on activated T cells, but it has also been recently detected on dendritic cells (DCs) (6–9). ICOS binds to ICOS ligand (ICOSL, also known as CD275, B7h, or B7H2), which is expressed by both hematopoietic and non-hematopoietic cells. Indeed, ICOSL is not only constitutively expressed by B cells, macrophages, and DCs, but it is also present in vascular endothelial cells (ECs), epithelial cells, fibroblasts, and different types of tumor cells (10). The cell distribution of ICOSL suggests that the ICOS/ICOSL complex may play an important role not just in Th cell activation within lymphoid organs but also in the regulation of T-cell functions in inflamed peripheral tissues.

Th costimulation through CD28 induces the secretion of large amounts of interleukin-2 (IL-2), a cytokine essential for the clonal expansion of naïve T cells. In contrast, IL-2 expression levels are weakly increased following ICOS costimulation, which instead leads to enhanced production of IL-10 (11, 12). The observation that ICOS expression is induced by CD3/TCR signaling and further enhanced upon CD28 costimulation and IL-2 production suggests that some of the effects triggered by CD28 are required for ICOS activity (11–13). Moreover, the ICOS-mediated costimulation can work in synergy with those mediated by other costimulatory molecules, such as 4-1BBL, and CD70 (11, 12).

It is well established that ICOS plays a key role in the differentiation of regulatory T cells (Tregs), Th17, and T follicular helper cells (TFHs) (14–16). In addition, ICOS induces CD40L (CD154) expression in T cells and regulates T cell/B cell interactions by increasing the production of immunoglobulin (Ig) M and IgG in B cells, favoring the formation of germinal centers. Fittingly, ICOS deficiency causes common variable immunodeficiency (17–21).

Substantial differences in the downstream effects of ICOS costimulation have been reported in humans vs mice. In particular, ICOS triggering induces the secretion of large amounts of IL-4 in mice, whereas it mainly leads to the production of interferon-γ (IFN-γ) in humans. These two distinct effects appear to involve Th2 and Th1 cells, respectively (22–25). These differences might be partly due to the ability of human but not murine ICOSL to weakly interact with CD28 and cytotoxic T-lymphocyte antigen 4 (CTLA4), which inhibits T cell functions through a binding site different from that used by ICOS (26).

We have previously shown that CD3/ICOS costimulation of human naïve Th cells elicits different responses depending on the cytokine milieu: *i)* in the presence of IL-2, it mainly promotes IFN-γ secretion; *ii)* in the absence of IL-2, it stimulates production of IL-10 and transforming growth factor-β (TGF-β); and *iii)* in the presence of TGF-β+IL-1β, it induces the secretion of IL-17A. Under the latter condition, we also found that ICOS costimulation primarily induces the secretion of IL-17A and IL-10, whereas CD28 costimulation leads to secretion of IL-17F and IL-9 (11, 27).

ICOS/ICOSL interaction triggers bidirectional signals through either receptor, also modulating the response of ICOSL-expressing cell *via* a mechanism known as reverse signaling (28). In mice, reverse signaling through ICOSL leads to the production of IL-6 by DCs, thereby causing their activation (29). In human DCs, endothelial cells (ECs), and tumor cell lines, ICOSL signaling inhibits adhesiveness and migration; in human DCs, it also modulates cytokine secretion and promotes antigen cross-presentation, whereas in osteoclasts it hampers bone homeostasis. Moreover, it inhibits tumor growth and metastatization (30–35).

A previous micro-array analysis comparing the signature pathways in total CD4^+^ T cells following costimulation with ICOS, CD28, or CTLA-4 (36) has shown a strong similarity in gene expression changes induced by CD28 and ICOS, suggesting that their unique functional properties may result from differential expression of only a few genes (*i.e.*, IL-2 and IL-9). Additionally, data from the literature and bioinformatics databases obtained by RNA-Seq have characterized the expression profile of resting naïve CD4^+^ T helper cells vs resting memory CD4^+^ T cells or naïve CD4^+^ T cells after being induced to differentiate to different Th effector phenotypes in the presence of polarizing cytokines (37–39). However, no data are available regarding the early phases of naïve CD4^+^ T cells activation mediated by the first signal alone (CD3) or following ICOS or CD28 costimulation in the absence of polarizing cytokines. Thus, taking advantage of RNA-Seq-based global transcriptome profiling, here we have characterized the transcriptome profile of human naïve CD4^+^ T cells during the initial phases of activation with anti-CD3 mAb alone (first signal) or in combination with soluble recombinant forms of the physiological ligands of CD28 or ICOS (second signal) (*i.e.*, B7.1-Fc and B7h-Fc, respectively) in the absence of polarizing cytokines.

Altogether, our findings indicate that CD28- and ICOS-mediated costimulatory signals elicit distinct sets of immunological and immunometabolic genes. The implications of our findings in T cell homeostasis is discussed in the context of past and present literature.

## Material and methods

### Cells

Peripheral blood mononuclear cells (PBMCs) were isolated from buffy coats, kindly provided by the local blood transfusion service (Novara, Italy) upon informed consent, through Ficoll-Hypaque (Lympholyte-H; Cedarlane Laboratories Ltd., Burlington, ON, Canada; cod. CL5020) density centrifugation. Naïve CD4^+^ T cells were purified with EasySep™ Human naïve CD4^+^ T cells Negative Selection Kit (STEMCELL Technologies, Vancouver, BC, Canada; cod. 19155). This approach provided > 97% CD4^+^/CD45RA^+^/CD45RO^-^ (eBioscience, San Diego, CA, USA; cod. 17-0048-42; 12-0458-42; 11-0457-42) cells, as judged by direct immunofluorescence and flow cytometry (BD Biosciences, San Diego, CA). Cells derived from separate donors and were not pooled. From each donor, cells were divided into 10 wells of a 96 well plate, using 10 wells for each experimental condition (CD3, CD3+ICOS, CD3+CD28). After 3 days, cells were pooled by condition, to perform the RNA extraction. The use of buffy coats was approved by the local Ethics Committee (No. CE 88/17), and the study was conducted in accordance with the Declaration of Helsinki.

### Naïve CD4^+^ T cell activation

Round-bottom 96-well plates were coated with 100 μl of anti-CD3 mAb (OKT3, 10 μg/ml) overnight at 4°C. To stimulate ICOS or CD28, plates were washed with PBS and further coated with B7h-Fc (5 μg/ml; Bio-Techne, Minneapolis, MN, US; cod. 165-B7) or B7.1-Fc (5 μg/ml; Bio-Techne; cod. 140-B1) for 2 h at room temperature. Plates were then washed with PBS, and purified naïve CD4^+^ T cells were seeded in ten wells for each condition at 10^5^ cells/well in 200 μl of RPMI 1640 (Invitrogen, Burlington, ON, Canada; cod. 61870010) plus 10% FBS (Invitrogen; cod. 10270106) for 3 days.

### ELISA assay

To analyze IL-2 secretion upon cell activation, supernatants were collected at day 3 of culture and standard enzyme-linked immunosorbent assays (ELISA) was used to evaluate secretion of IL-2 (Biolegend, San Diego, CA, USA; cod. 431804) following the manufacturer’s instructions

### RNA-Seq sample preparation

Total RNA was isolated using TRIzol reagent (Invitrogen), following the manufacturer’s instructions. RNA quality was assessed using an Agilent 2100 Bioanalyzer. All samples had an RNA integrity number (RIN) ≥ 9. For RNA-Seq single-end library preparation, approximately 2 μg of total RNA were subjected to poly(A) selection, and libraries were prepared using the TruSeq RNA Sample Prep Kit (Illumina), following the manufacturer’s instructions. Sequencing was performed on Illumina platforms. All experiments were performed in biological triplicates.

### RNA-Seq and bioinformatics data analyses

Reads were mapped to the *Homo sapiens* hg19 reference genome using TopHat v2.0.10 (40). For data analysis, gene counts were computed using htseq-count and the GENCODE v24 gene annotation. Differential expression analysis was performed using DESeq2 R Package (41). Only genes with |log2FoldChange| ≥ 1, FDR < 0.05 and RPKM ≥ 1 in at least one of the analyzed conditions were considered for downstream analysis.

The R statistical environment was used for further statistical analysis (42). Principal Component Analysis (PCA) plot was performed using the prcomp package on the matrix of expression data. Heatmaps were computed using the pheatmap package. Volcano plots were computed using scatter plot function.

The characterization of activated pathways in modulated genes was computed using Ingenuity Pathway Analysis (IPA) (QIAGEN Inc., https://www.qiagen.com/us/products/discovery-and-translational-research/next-generation-sequencing/informatics-and-data/interpretation-content-databases/ingenuity-pathway-analysis/).

RNA-Seq data have been deposited in the Gene Expression Omnibus database with the dataset identifier GSE191040.

### Real-time RT-PCR

Total RNA was isolated from purified naïve CD4^+^ T cells activated with anti-CD3 mAb alone, plus B7h-Fc or B7.1-Fc at day 3 using TRIzol reagent (Invitrogen; cod. 15596018). RNA (1 μg) was retrotranscribed using a QuantiTect Reverse Transcription Kit (Qiagen, Hilden, Germany; cod. 205313). NEBL (Hs01067284_m1), SHC4 (Hs00736166_m1), IL1RL1 (Hs00249384_m1), TMCC2 (Hs01099575_m1), FBXO15 (Hs00380856_m1), PALLD (Hs00363101_m1), HOPX (Hs04188695_m1), CDO1 (Hs01039954_m1), NEAT1 (Hs01008264_s1), IL23R (Hs00332759_m1), IL26 (Hs00218189_m1), SOCS3 (Hs02330328_s1), and ICOS (Hs00359999_m1) mRNA expression levels were assessed through Assay-on Demand (Applied Biosystems, Foster City, CA). GAPDH (Hs99999905_m1) was used to normalize the cDNA amounts. Real-time PCR was performed using the CFX96 System (Bio-Rad Laboratories, Hercules, CA, USA) in duplicate for each sample in a 10 μl final volume containing 0.5 μl of diluted cDNA, 5 μl of TaqMan Universal PCR Master Mix (Applied Biosystems; cod. 4369016), and 0.5 μl of Assay-on Demand mix. The thermocycler parameters were 95°C for 10 min, followed by 45 cycles of 95°C for 15 s and 60°C for 1 min. The results were analyzed with a ΔΔ threshold cycle method, and the relative gene expression was expressed as fold increase/decrease over anti-CD3 mAb alone samples. Samples used for PCR validations were different than those used for the RNA-Seq analysis.

### Statistical analysis

The paired T-test was used to compare differences in real-time PCR experiments using GraphPad Instat Software (GraphPad Software, San Diego, CA). *P*-values < 0.05 were considered statistically significant.

## Results

**Figure 1:**
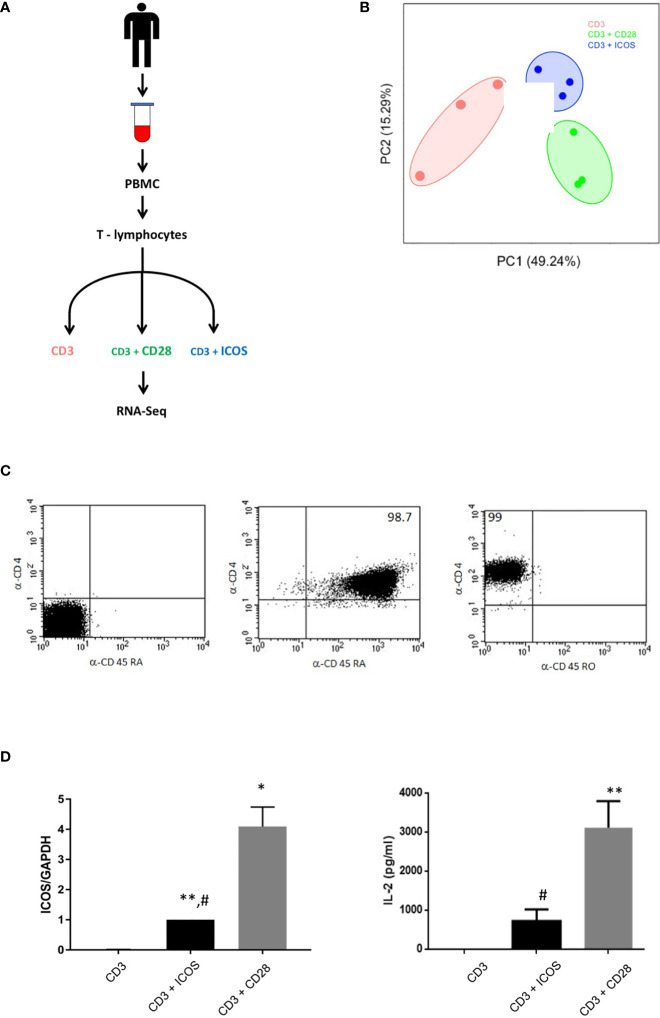
CD3+ICOS and CD3+CD28 costimulation experimental design. **(A)** Schematic representation of the experimental design. **(B)** Principal component analysis (PCA) plot of the mRNA-seq samples. **(C)** Cytofluorimetric analysis of CD4, CD45RA, and CD45RO expression on CD4 naïve T cells immediately after the purification. Data are from one representative experiment. The percentage of the purity is indicated within the quadrants **(D)** Left Panel shows ICOS mRNA validation by real-time PCR while right panel shows IL-2 production by ELISA. Data are normalized for the expression of each gene under anti-CD3 treatment condition *P < 0.05, **P < 0.01 vs anti-CD3; #P < 0.05, vs anti-CD3+CD28.

Purified T CD4^+^ naïve cells were stimulated *in vivo* with anti-CD3 mAb in the absence (CD3 group) or presence of the B7.1-Fc fusion protein, which triggers CD28, (CD3+CD28 group) or the ICOS ligand B7h-Fc (CD3+ICOS group) (**Figures 1A, C**). RNA-Seq was performed after 3 days of culture. **Figure 1B** shows the result of the PCA plot analysis performed on the RNA-Seq data. Only results of the principal components 1 and 2 are reported. The three replicates of each group cluster with relative low distance and do not overlap, indicating a significant structural similarity between their gene markers and comparable results among samples belonging to the same experimental group. **Figure 1D** (left panel) shows the real-time PCR validation of ICOS gene expression under the three different experimental conditions and **Figure 1D** (right panel) shows the secretion of IL-2, measured by ELISA, marking T cell activation. Both report effective costimulation by ICOS and CD28 but with CD28 exerting stronger effects, as expected.

### Different costimulatory patterns drive specific transcriptional responses

**Figure 2:**
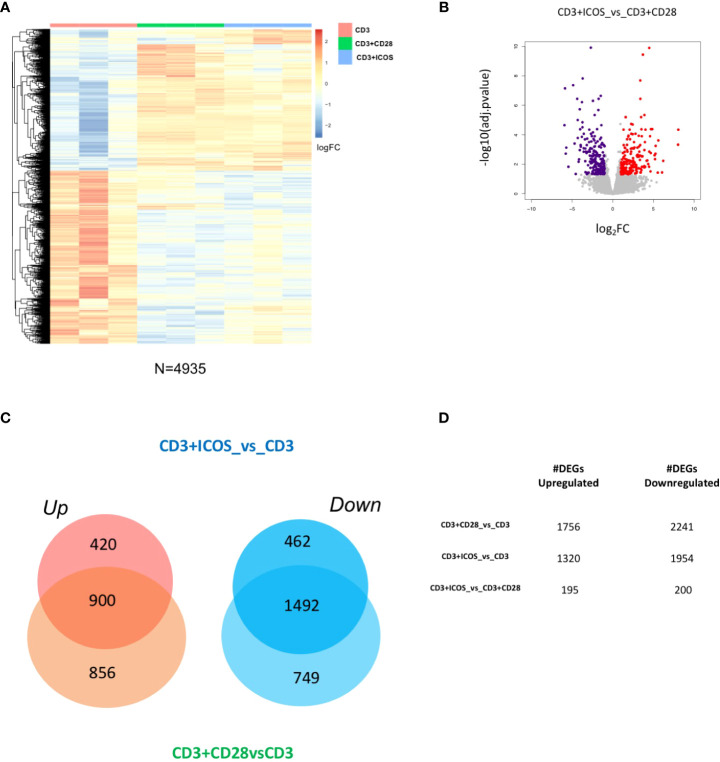
CD3+ICOS and CD3+CD28 costimulations drive global transcriptional changes. **(A)** Heatmap showing the unsupervised hierarchical clustering of differentially expressed genes (DEGs) from the three conditions described in the legend to **Figure 1A**. **(B)** Volcano plot displaying identified DEGs in the CD3+ICOSvsCD3+CD28 group. Red dots represent upregulated genes, while violet dots represent downregulated genes. **(C)** Venn diagram reporting the portion of DEGs found modulated in the CD3+ICOSvsCD3 and CD3+CD28vsCD3 comparison groups. UP = upregulated genes; DOWN = downregulated genes. **(D)** Table reporting the total number of DEGs according to each comparison group.

To identify the transcriptional responses associated with each costimulation, we performed comparative transcriptome analysis of the following experimental conditions: CD3+CD28vsCD3, CD3+ICOSvsCD3, and CD3+ICOSvsCD3+CD28. We found 4935 differentially expressed genes (DEGs, **Supplementary Table 1**) in at least one comparison (|log2FoldChange| ≥ 1, FDR < 0.05; **Figure 2A**). The log2 fold changes and the -log10(adj.pvalue) of all upregulated and downregulated DEGs (195 and 200, respectively) in CD3+ICOSvsCD3+CD28 are depicted in **Figure 2B**. The proportion of genes either up- or downregulated in the CD3+CD28vsCD3 and CD3+ICOSvsCD3 comparison groups are represented in **Figure 2C**. The Venn diagram indicates that, among the upregulated genes, 900 genes are shared by the two groups, while 856 and 420 are specific for the CD3+CD28 and CD3+ICOS group, respectively. Among the downregulated genes, 1492 are in common, whereas 749 and 462 are unique to the CD3+CD28 and CD3+ICOS group, respectively. **Figure 2D** shows the numbers of upregulated and downregulated genes for each comparison group.

Thus, human CD4^+^ Th cells display specific transcriptional activities in response to different costimulatory signals.

### Validation of high-throughput RNA-Seq results

We next sought to validate our mRNA-seq results at the single gene level. Since ICOS and IL-2 are DEGs detected by the RNA-seq analysis, a first validation was obtained by assessment of ICOS expression by real time PCR and IL-2 secretion by ELISA, confirming the expression pattern obtained by RNA-seq (**Figure 1D**).

**Figure 3:**
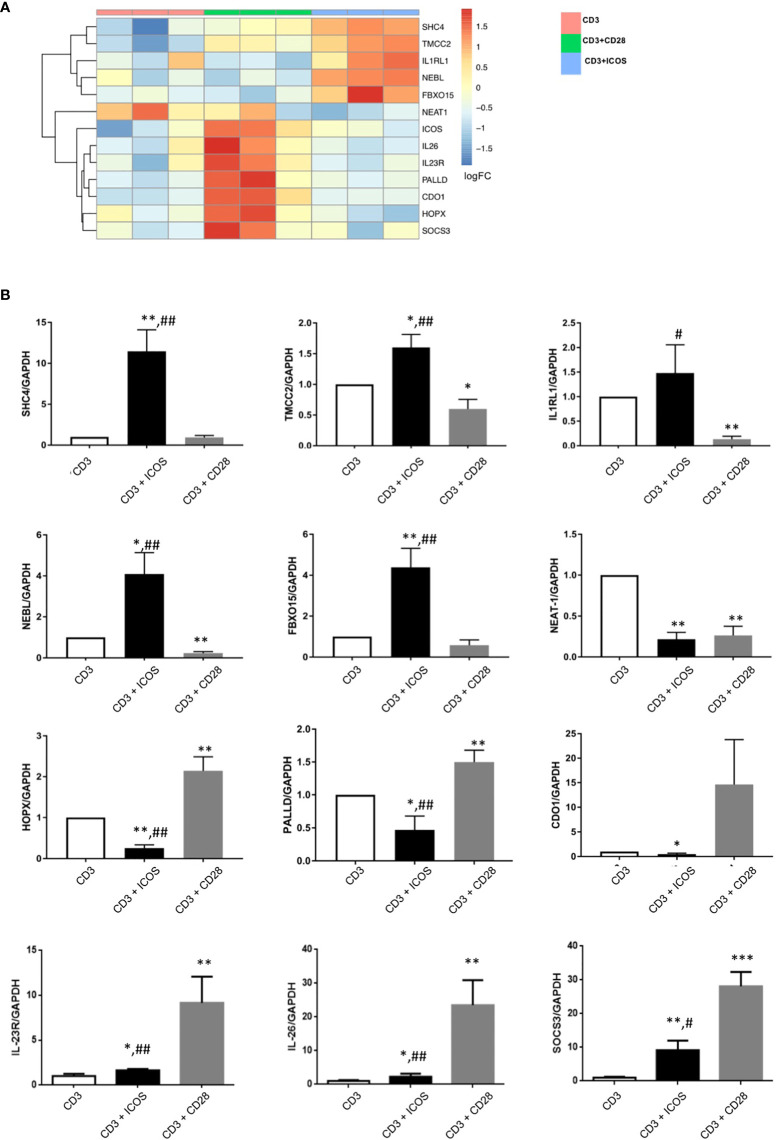
Validation of selected DEGs by real-time PCR. **(A)** Heatmap showing unsupervised hierarchical clustering of the ICOS gene and a panel of 12 DEGs randomly selected for gene expression validation by real-time PCR. **(B)** Results of real-time PCR validation of the 12 randomly selected genes analyzed in T CD4+ naïve cells from 3 new donors. Data are normalized for the expression of each gene under the anti-CD3 treatment condition; *P < 0.05, **P < 0.01, *** P < 0.0001 versus anti-CD3; #P < 0.05, ##P < 0.01 vs anti-CD3+CD28.

To extend this validation, we repeated the activation experiment on T CD4+ naïve cells purified from 3 new donors and analyzed the expression of 12 randomly selected DEGs (SHC4, TMCC2, IL1RL1, NEBL, FBXO15, NEAT1, HOPX, PALLD, CDO1, IL-23R, IL-26, and SOCS3). The heatmap showing the unsupervised hierarchical clustering of mRNA-seq expression levels for this selection of DEGs is represented in **Figure 3A**. The real time PCR analyses on the new samples confirmed the expression patterns obtained by RNA-Seq (**Figures 3B**). Therefore, validation was obtained for all 14 genes analyzed in the two experiments (i.e ICOS and IL-2 in the first experiment and the 12 randomly selected genes in the second experiment).

### ICOS-mediated costimulation highlights differentially modulated functional pathways

**Figure 4:**
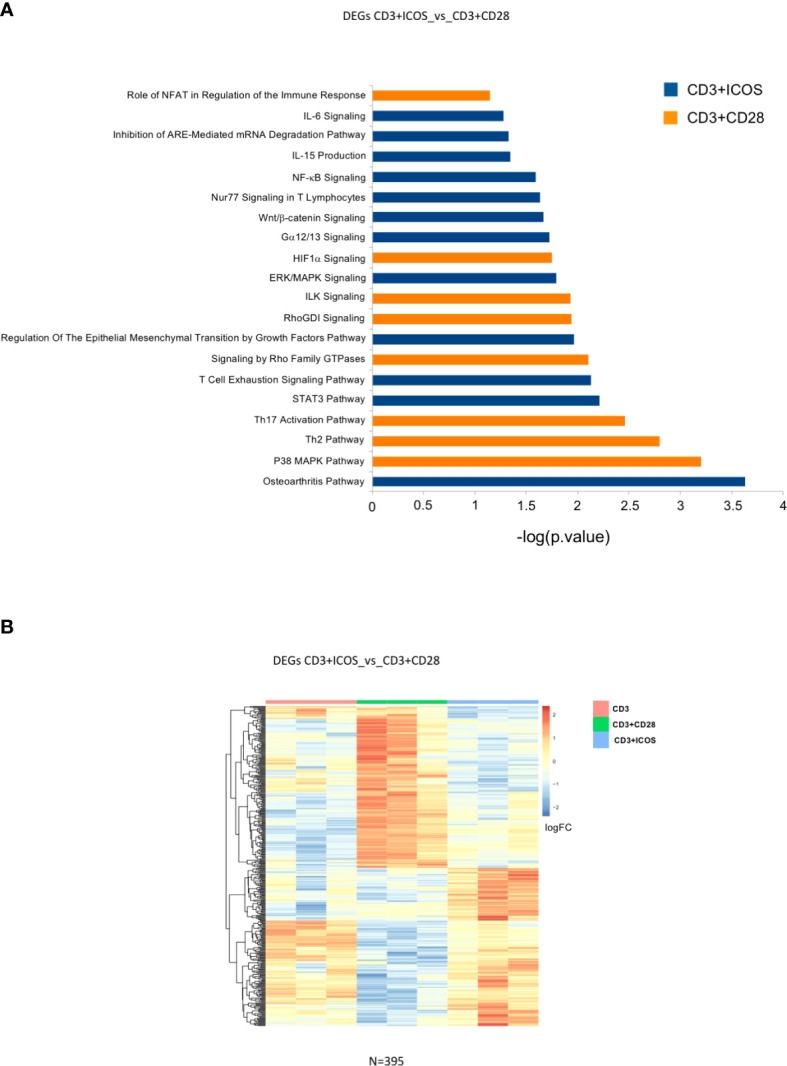
Modulated genes and enriched pathways in the CD3+ICOSvsCD3+CD28 group. **(A)** Selected enriched pathways in the CD3+ICOSvsCD3+CD28 group as predicted by Ingenuity Pathway Analysis (IPA). Blue bars: enriched in CD3+ICOS; Orange bars: enriched in CD3+CD28. **(B)** Heatmap of differentially expressed genes in CD3+ICOSvsCD3+CD28 comparison.

We next sought to determine DEG functions and identify possible biological networks in which they may be involved, with a particular focus on the effects mediated by CD3+ICOS stimulation. To this end, DEGs modulated by each costimulation were imported into the IPA software. **Figure 4** shows the selected activated pathways of genes identified by IPA for both CD3+ICOS and CD3+CD28 costimulation. **Figure 4A** shows the functional annotation of DEGs in the CD3+ICOSvsCD3+CD28 group, while **Figure 4B** illustrates the unsupervised hierarchical clustering of these genes. The pattern suggests that ICOS and CD28 play distinct roles in the regulation of several key immunological pathways, such as pathways involved in cytokine production and signaling, immune receptor signaling, and Th polarization (**Figures S1A, B**). In particular, ICOS-mediated costimulation activates several pathways involved in known aspects of ICOS function. A pivotal role is played by pathways related to IL-10 activity, as judged by a significant enrichment of the biological processes “STAT3”, “T cell exhaustion signaling”, and “Nur77 signaling in T cells”. Indeed, STAT3 plays a crucial role in IL-10R-mediated signaling, T cell exhaustion involves IL-10 production, and Nur77 is involved in T cell exhaustion and tolerance as well as Treg function (43–45). Other noteworthy ICOS-related pathways are “IL-6 signaling” and “IL-15 production” pathways. IL-6 plays a role in differentiation of Th17 cells and function of TFH cells, which are Th cell subsets involving ICOS function, and triggers the STAT3 pathway through IL-6R (46). IL-15 shares many activities with IL-2, and may partly overcome the poor ability of ICOS to induce IL-2 secretion compared to CD28 (47). Notably, the signature “Regulation of the EMT by growth factors pathway” is in good agreement with the observation that activation of ICOS/ICOSL signaling inhibits EMT in tumor cells lines (31). However, the highest scores were reached by the osteoarthritis (OA) pathway for ICOS and by the p38 MAPK pathway for CD28.

**Figure 5:**
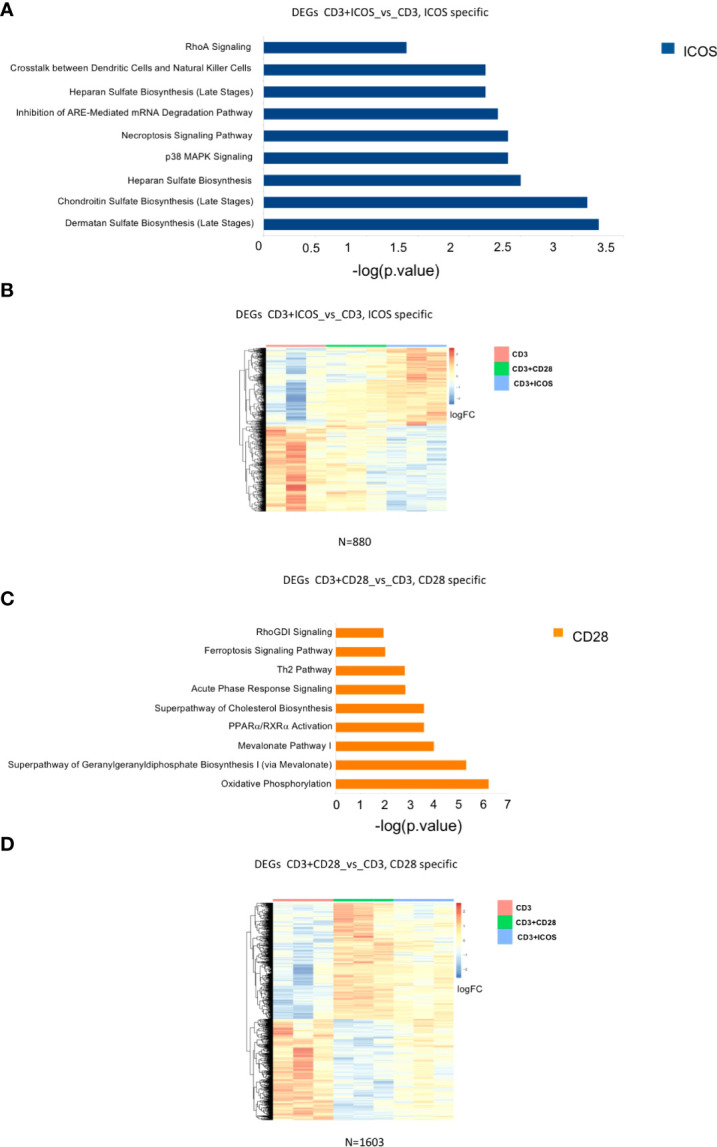
Modulated genes and enriched pathways in CD28 and ICOS specific gene signatures. **(A)** Selected enriched pathways in CD3+ICOSvsCD3; “ICOS-specific” comparison as predicted by Ingenuity Pathway Analysis (IPA). Only the pathways activated by ICOS-modulated genes are shown (blue bars). **(B)** Heatmap of DEGs in CD3+ICOSvsCD3; “ICOS-specific” comparison. **(C)** Selected enriched pathways in CD3+CD28vsCD3; “CD28-specific” comparison as predicted by IPA Ingenuity. Only pathways activated by CD28-modulated genes are shown (orange bars). **(D)** Heatmap of differentially expressed genes CD28vsCD3; “CD28-specific” comparison.

We next sought to identify a set of DEGs that would only be specific for CD3+ICOS costimulation. For this purpose, we selected only those genes that were modulated in the CD3+ICOSvsCD3 group but remained unchanged in the CD3+CD28vsCD3 group. This approach allowed us to define a set of 880 genes, termed “ICOS-specific”, which were subjected to functional analysis by IPA (**Figure 5A**). The relative heatmap is reported in **Figure 5B**.

We also defined a group of DEGs comprising only those genes modulated in the CD3+CD28vsCD3 group but unaltered in the CD3+ICOSvsCD3 group. We identified a set of 1603 DEGs, termed “CD28-specific”, which were subjected to a functional analysis by IPA as described above (**Figure 5C**). The heatmap of the genes modulated in this comparison group is depicted in **Figure 5D**.

Overall, the functional annotations of ICOS- and CD28-specific DEGs reveal distinct signaling pathways activated by CD3+ICOS and CD3+CD28, highlighting a divergent regulation of immunometabolism. Although both ICOS and CD28 appear to alter T cell glycosaminoglycan metabolism with a similar trend, ICOS seems to exert a stronger effect. Indeed, ICOS promotes the expression of isoform 3 and 10 of carbohydrate sulfotransferase (CHST), while downmodulates isoform 15. It also promotes the expression of heparan sulfate glucosamine 3-O-sulfotransferase 1 (HS3ST1), N-deacetylase and N-sulfotransferase 1 (NDST1) and Notum (cleavage of GPI anchored heparan sulfate proteoglycans), while it decreases dermatan sulfate epimerase (DSE) and chondroitin sulfate N-acetylgalactosaminyltransferase 2 (CSGALNACT2).

Altogether, these changes suggest an active remodeling of surface proteoglycans induced selectively by ICOS (**Figure S1C**).

With regard to CD28-specific metabolic pathways, the difference with ICOS is mainly quantitative. Indeed, both CD28 and, to a lesser extent, ICOS promotes transcription of mitochondrial respiratory chain components, such as several complex I subunits (NDUFB4, NDUFA8, NDUFA6, NDUFS4, NDUFB8, NDUFV2, NDUFA1, NDUFB6, and NUDUFB3), complex II (SDHB), complex III (UQCRH), cytochrome c isoform 1 (CYC1), and both central and accessory complex IV subunits (COX5B; COX6C; COX7B and COX7A2). Interestingly many of these proteins are involved in the formation of respiratory supercomplexes, suggesting a global reorganization of the respiratory chain (48, 49) (**Figure S1D**). Likewise, the cholesterol metabolism pathway appears to be induced by both ICOS and, to a greater extent, CD28, but only the CD28 effect is strong enough to reach significance. Finally, our results show the induction of enzymes related to fatty acid metabolism (ACAT2 and ACAA2) and cholesterol biosynthesis (MSMO1, MVD, MVK, HMGCS1, IDI1 and FDPS), suggesting an increased utilization of fatty acid and a parallel activation of the mevalonate/cholesterol biosynthetic pathway (**Figure S1E**).

## Discussion

By taking advantage of the higher sensitivity of RNA-Seq over microarray technology, here we have compared the transcriptome profiles of human naïve CD4^+^ T cells following costimulation of either CD3+ICOS or CD3+CD28. Our results derived from two different comparisons show that distinct sets of immunological and immunometabolic genes are differentially regulated by ICOS- and CD28-mediated costimulatory signals, suggesting that the concerted modulation of multiple downstream pathways by costimulatory molecules of the CD28 family plays a crucial role in T cell homeostasis.

We initially looked for DEGs by directly comparing the effects of two costimulations (*i.e.*, CD3+ICOS vs CD3+CD28), which detected 395 differentially modulated genes. Functional annotation by IPA shows that ICOS-mediated costimulation activates several pathways involved in known aspects of ICOS function, such as those related to IL-10, IL-6, and IL-5 function. However, the highest IPA score was obtained by the “osteoarthritis pathway”—*i.e.*, genes involved in the OA microenvironment (**Figure S1A**). This finding is quite interesting in light of the recent view that OA, normally regarded as a non-inflammatory disease of the joints caused by mechanical stress resulting in joint cartilage destruction, may indeed exhibit infiltration of synovial membranes by inflammatory cells—even T cells—, suggesting that T cell-mediated immune responses may play a role in this disease. In particular, a key role may be played by CD4^+^ T cells and especially Th1, Th17, which accumulate in the synovial fluid and/or membranes of OA patients. Moreover, a role in OA development has been suggested for TFH cells whose number, together with IL-21 production, is increased in the blood of OA patients and positively correlates with disease activity (50, 51). Overall, our data are consistent with the notion that Th1, Th17, and TFH cells are particularly related to ICOS function in humans.

With regard to the pathways activated by CD28 costimulation, the highest IPA score was recorded for the p38 MAPK signaling pathway (**Figure S1B**), which is in line with the well-established involvement of this pathway in CD28-dependent T cell activation. A modulation of the p38 pathway also emerges upon ICOS treatment, but the list of modulated genes differs from that modulated by CD28 confirming the notion of differential signaling between the two. Importantly, the detection of the “Th2 pathway” is in keeping with the ability of CD28, but not ICOS, to support differentiation of Th2 cells in humans. By contrast, the detection of the “Th17 activation pathway” may highlight a stronger effect of CD28 compared to ICOS in supporting Th17 cells, since both CD28 and ICOS support Th17 differentiation, albeit with some differences (27).

Along the same lines, the hypoxia-inducible factor-α (HIF-α) pathway is differentially enhanced in the CD3+CD28vsCD3+ICOS group. HIF induction during naive T cell activation is strongly dependent on Phosphoinositide 3-kinase (PI3-K)/mTOR and CD28 signaling (52). Since both CD28 and ICOS activate PI3K, enhanced induction by CD28 might stem from the different activation of PI3K due to distinct expression levels of CD28 and ICOS.

In the second phase of our study, we assessed possible differences in gene expression between ICOS- and CD28-mediated costimulation by indirectly comparing the genes modulated by each pathway with those regulated by CD3 signaling (CD3 alone vs CD3+ICOS or CD3+CD28). We show that 880 genes are specifically modulated by ICOS and 1603 genes by CD28. Functional annotation of these DEGs pinpoints to differences mostly related to metabolic pathways, a quite intriguing finding given the emerging data pointing to cellular metabolism as a key player in T cell activation and function, a process defined as immunometabolism. In particular, the functional analysis of the 880 genes specifically modulated by ICOS mainly detected genes involved in glycosaminoglycan biosynthesis (**Figure S1C**), including biosynthesis of dermatan sulphate, chondroitin sulphate, and heparan sulphate. Since glycosaminoglycan biosynthesis is involved in tissue repair, this observation is in good agreement with the data of our first analysis assessing the effects of ICOS- vs CD28-mediated costimulation, highlighting pathways involved in late phases of the immune response (53, 54). Fittingly, studies in mice have shown that ICOS signaling is involved in wound healing (55, 56).

Surface proteoglycans act at as concentrators of chemokines and cytokines, such as SDF-1 and IL-12, and modulate their presentation to receptors (57, 58). Interestingly, heparan sulphate mimetics perturbs T cell activation and differentiation, and blocking heparan sulphate biosynthesis by NDST1/2 silencing in T cells increases the proliferative response to weak activation stimuli (59, 60). These data suggest that increased NDST1 expression and modulation of heparan sulphate metabolizing enzymes may contribute to the weaker proliferative response of T cells following ICOS-mediated costimulation in comparison with what observed following CD28-mediated costimulation. This would also be in agreement with the notion that ICOS-mediated costimulation supports effector T cell responses, whereas CD28 signaling is mainly involved in clonal expansion of T cells (10).

Among other glycosaminoglycans receptors, one cannot fail to mention CD44, which modulates T cell activation and function and enhances Treg activity by interacting with several ligands including hyaluronan and chondroitin sulphate (53, 61). Moreover, CD44 is subjected to a wide array of post-translational carbohydrate modifications, including glycosaminoglycan side chain additions, which can have profound effects on CD44 binding function (62). It is also worth mentioning that another ligand of CD44 is osteopontin (OPN), which is an ICOSL ligand as well. ICOS and OPN bind to ICOSL through a different binding site and elicit different, often opposite, functional effects (35). Therefore, by activating glycosaminoglycan synthesis, ICOS might also influence the balance within the CD44/OPN/ICOSL/ICOS network.

Incidentally, molecules involved in the crosstalk between DC and Natural Killer (NK) cells are differentially expressed by CD3+ICOS-activated cells. Interestingly, our recent results show expression of both ICOS/ICOSL in either population, with clear consequences for NK function (63).

Functional analysis of the 1603 genes specifically modulated by CD28 mainly detected genes involved in the oxidative phosphorylation pathway, in particular components of the mitochondrial respiratory chain. CD28 also induces genes involved in fatty acid utilization and the mevalonate/cholesterol biosynthetic pathway (**Figures S1 D, E**). This finding fits with the notion that T cell activation is not only supported by aerobic glycolysis and oxidative phosphorylation, but it is also accompanied by a switch to anaerobic glycolysis, allowing fast production of Adenosine triphosphate (ATP) and availability of a carbon source for the macromolecular synthesis required for clonal expansion and differentiation (64, 65). Interestingly, a recent work has proposed that ICOS signaling may play a role in the maintenance of a constitutive glycolytic phenotype in long-lived TFH cells (66).

The modulation of respiratory chain supercomplexes by both CD28 and, to a lesser extent, ICOS is, presumably, a strategy to optimize electron flux to provide ATP for cell proliferation and effector functions (67). Indeed, it is well known that resting T helper cells are characterized by oxidative metabolism, while their activation *via* CD3+CD28 costimulation increases glucose uptake and mitochondrial Reactive Oxygen Species (ROS) production. In these cells, available data indicate a role of complex III-dependent mitochondrial ROS in NFAT activation (68). Furthermore, increased mitochondrial activity is necessary for proliferation, while cytokine production is less sensitive to mitochondrial inhibitors (69). Accordingly, we show that ICOS costimulation results in a weaker induction of the expression levels of mitochondrial respiratory chain components compared to those seen upon CD28 costimulation.

A differential balance between the aforementioned metabolic pathways also appears to be crucial for the differentiation of different types of Th and Treg cells. In particular, mevalonate seems to enhance the differentiation and suppressive activity of Treg cells through the TGF-β signaling pathway. The mevalonate pathway is also important in the synthesis of cholesterol and nonsterol isoprenoids. Cholesterol plays a role in ferroptosis, also highlighted by our analysis. Non-sterol isoprenoids include geranylgeranyl pyrophosphate (GGPP)—evidenced by our analysis—, which is required for T cell survival and function. Moreover, GGPP enhances IL-2 production and Foxp3 Treg cell development and function (70, 71).

Interestingly, several genes emerging as being regulated by CD28 during lipid metabolism, such as ACAA2, HMGCS1, and IDI1, are targets of PPARα (72, 73). Thus, their increased expression may depend on activation of the PPARα pathway, directly or indirectly, through the induction of SREBP. Among the CD28 induced genes, farnesyl diphosphate synthase (FDPS) is of particular interest, being a key regulator of Vγ2Vδ2 T cells activation (74).

In conclusion, these data suggest that ICOS- and CD28-mediated costimulations play distinct roles during the activation of naïve T cells by modulating distinct sets of immunological and immunometabolic genes. Given the different effects of ICOS and CD28 on Th cell costimulation, the modulation of distinct sets of immunological genes was expected, albeit not detected by previous microarray analyses. Modulation of immunometabolic genes by CD28-mediated costimulation was also expected given the literature describing the immunometabolic effect of T cell activation. In this regard, the activation of mevalonate pathways underscores the role of CD28 in Treg activity. However, the effect of ICOS-mediated costimulation on several glycosaminoglycan biosynthesis pathways was unexpected and paves the way to a novel research field on ICOS-mediated costimulation.

## Data availability statement

The datasets presented in this study can be found in online repositories. The names of the repository/repositories and accession number(s) can be found below: https://www.ncbi.nlm.nih.gov/geo/, GSE191040.

## Ethics statement

The studies involving human participants were reviewed and approved by the local Ethics Committee (No. CE 88/17), and the study was conducted in accordance with the Declaration of Helsinki. The patients/participants provided their written informed consent to participate in this study.

## Author contributions

CG and EB performed the experiments (cell purification, cell stimulation, RNA and protein analyses) and analyzed the data; DI ran RNA-Seq experiment; DI, FF, DC and SZ performed bioinformatics analysis; CS, SO, DC, UD, FP, GB, JR, and SZ designed the study and analyzed the data. DC, FF, and UD wrote the manuscript. All authors contributed to the article and approved the submitted version.

## Funding

This work was supported by Fondazione CARIPLO (2014-0812) to SZ. and by the Associazione Italiana Ricerca sul Cancro (IG 20714 to UD and IG 20240 to SO, AIRC, Milano), and Fondazione Cariplo (2017-0535) to UD. DC acknowledge support by the Italian Ministry of University and Research program “Departments of Excellence 2018-2022”, AGING Project – Department of Translational Medicine, Università del Piemonte Orientale. Fondazione Umberto Veronesi, Milan, Italy supported EB.

## Acknowledgments

This work is dedicated to the memory of our colleague SZ. SZ was an extraordinary person, a great friend, a remarkable scholar and an unfailing mentor for our students. Her passion for life and research will always be an example. We miss her a lot.

## Conflict of interest

The authors declare that the research was conducted in the absence of any commercial or financial relationships that could be construed as a potential conflict of interest.

## Publisher’s note

All claims expressed in this article are solely those of the authors and do not necessarily represent those of their affiliated organizations, or those of the publisher, the editors and the reviewers. Any product that may be evaluated in this article, or claim that may be made by its manufacturer, is not guaranteed or endorsed by the publisher.

## Supplementary material

Supplementary Figure 1
**(A)** Heatmap showing unsupervised hierarchical clustering of genes modulated in the osteoarthritis (OA) pathway in the CD3+ICOSvsCD3+CD28 group. **(B)** Heatmap showing unsupervised hierarchical clustering of genes modulated in the p38 pathway in the CD3+ICOSvsCD3+CD28 group. **(C)** Heatmap showing unsupervised hierarchical clustering of glycosaminoglycans in the CD3+ICOSvsCD3 group, “ICOS_specific”. **(D)** Heatmap showing unsupervised hierarchical clustering of CD28-specific genes modulated in the oxidative phosphorylation pathway. **(E)** Heatmap showing unsupervised hierarchical clustering of CD28-specific genes modulated in the mevalonate/cholesterol pathway.

Ssupplementary Table 1 Complete list of DEGs.

## Abbreviations

APCs, antigen-presenting cells; ATP, Adenosine triphosphate; CTLA4, Cytotoxic T-Lymphocyte Antigen 4; DCs, Dendritic cells; DEGs, differentially expressed genes; ECs, vascular endothelial cells; GAGs, glycosaminoglycans; IFN, Interferon; Ig, immunoglobulin; IL, Interleukin; IPA, Ingenuity Pathway Analysis; mAb, monoclonal antibody; MHC, major histocompatibility complex; NK, Natural Killer; OA, osteoarthritis; OPN, osteopontin; PBMCs, Peripheral blood mononuclear cells; PCA, Principal component analysis; PI3-K, Phosphoinositide 3-kinase; ROS, Reactive Oxygen Species; TCR, T-cell receptor complex; TFHs, follicular helper cells; TGF-β, Transforming Growth Factor-β; Th, T helper cells; Tregs, regulatory T cells.
