# Supplementary figures and images for: Specific transcriptional programs differentiate ICOS from CD28 costimulatory signaling in human Naïve CD4+ T cells
Source: Front Immunol. 2022 Sep 5;13:915963. doi: 10.3389/fimmu.2022.915963 (PMC9484324; doi:10.3389/fimmu.2022.915963)

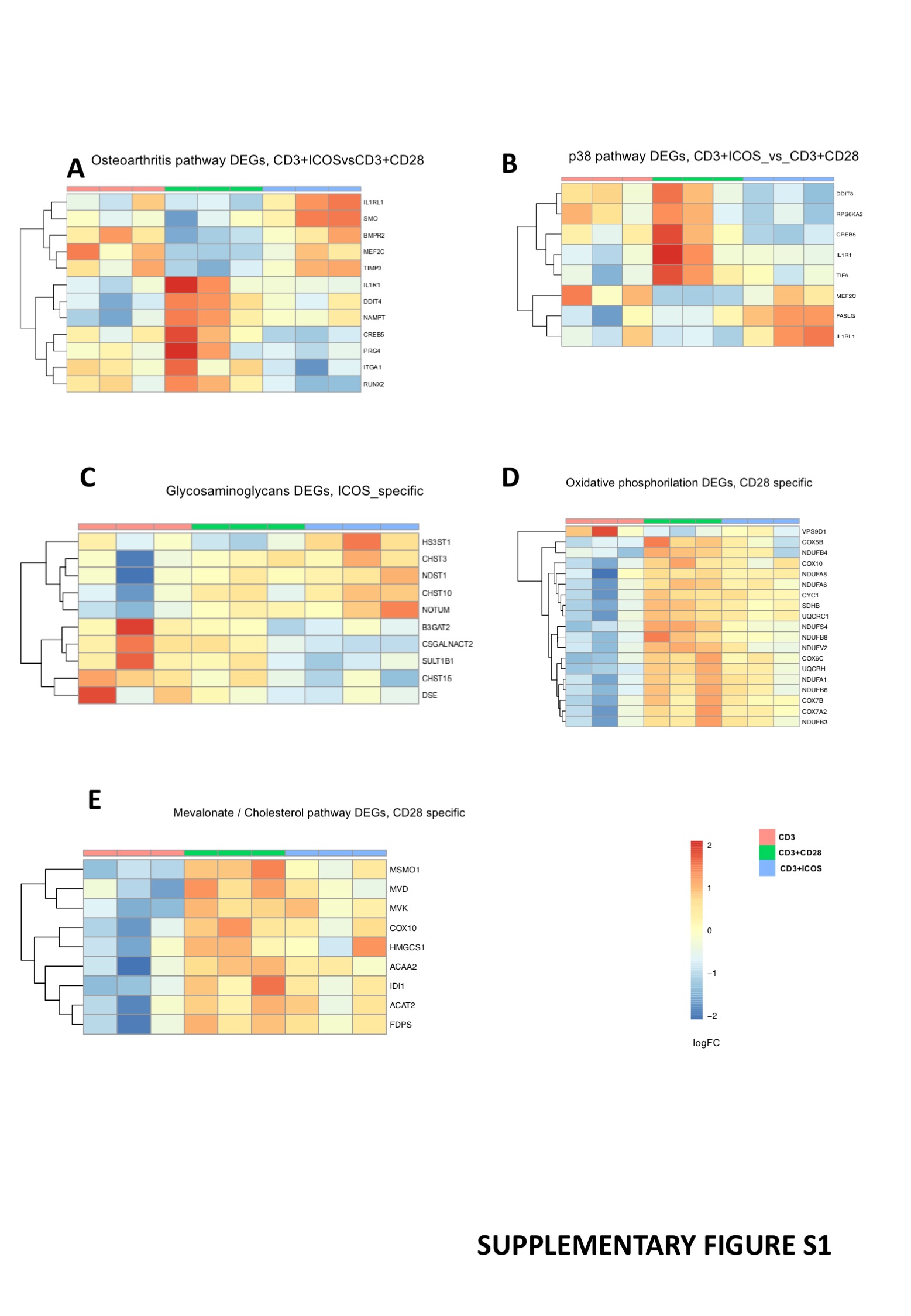

Supplement: Supplementary Figure 1 — (A) Heatmap showing unsupervised hierarchical clustering of genes modulated in the osteoarthritis (OA) pathway in the CD3+ICOSvsCD3+CD28 group. (B) Heatmap showing unsupervised hierarchical clustering of genes modulated in the p38 pathway in the CD3+ICOSvsCD3+CD28 group. (C) Heatmap showing unsupervised hierarchical clustering of glycosaminoglycans in the CD3+ICOSvsCD3 group, “ICOS_specific”. (D) Heatmap showing unsupervised hierarchical clustering of CD28-specific genes modulated in the oxidative phosphorylation pathway. (E) Heatmap showing unsupervised hierarchical clustering of CD28-specific genes modulated in the mevalonate/cholesterol pathway. [file Image_1.jpeg]
